# Ionic Liquids as Advanced Lubricant Fluids

**DOI:** 10.3390/molecules14082888

**Published:** 2009-08-04

**Authors:** María-Dolores Bermúdez, Ana-Eva Jiménez, José Sanes, Francisco-José Carrión

**Affiliations:** Grupo de Ciencia de Materiales e Ingeniería Metalúrgica, Departamento de Ingeniería de Materiales y Fabricación, Universidad Politécnica de Cartagena, Campus Muralla del Mar. 30202-Cartagena, Spain

**Keywords:** ionic liquids, surface interactions, tribochemistry, additives, nanotechnology

## Abstract

Ionic liquids (ILs) are finding technological applications as chemical reaction media and engineering fluids. Some emerging fields are those of lubrication, surface engineering and nanotechnology. ILs are thermally stable, non-flammable highly polar fluids with negligible volatility, these characteristics make them ideal candidates for new lubricants under severe conditions, were conventional oils and greases or solid lubricants fail. Such conditions include ultra-high vacuum and extreme temperatures. Other very promising areas which depend on the interaction between IL molecules and material surfaces are the use of ILs in the lubrication of microelectromechanic and nanoelectromechanic systems (MEMS and NEMS), the friction and wear reduction of reactive light alloys and the modification of nanophases.

## Introduction

**Figure 1:**
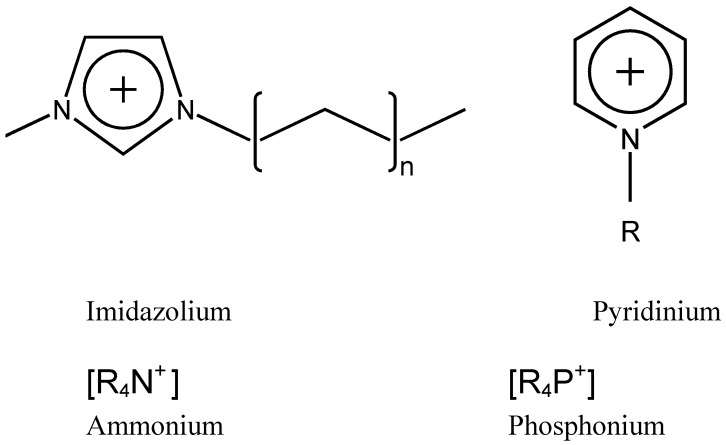
Some common IL cations used in lubrication and surface modification.

Salts with melting points lower than 100 ºC are called ionic liquids. In this work, we will refer mainly to room-temperature ionic liquids, which present melting points lower than room temperature. In what follows we will use the general abbreviation IL to refer to ionic liquids and room-temperature ionic liquids. ILs are usually composed of an organic cation, typically containing nitrogen or phosphorus, and a weakly coordinating anion. Some of the most common cations (Figure 1) are imidazolium, phosphonium, pyridinium and ammonium, while some common anions are BF_4_^-^; PF_6_^-^; CF_3_SO_3_^-^ and N(CF_3_SO_2_) _2_^-^. 

The possibility of tuning the chemical and physical properties by changing the anion-cation combination is a great opportunity to obtain task-specific ILs. Understanding their structural properties [1,2,3] is essential for a systematic design. 

Although some ILs can be distilled under vacuum at 200-300 ºC [4], they present a negligible vapour pressure. Other unique characteristics of ILs are their high polarity, high thermal stability, nonflammability, miscibility with water and with organic solvents and their electrochemical properties. This combination is responsible for their growing number of applications, mainly in chemical synthesis and extraction processes, but also as engineering fluids [5,6,7,8,9,10,11], in nanotechnology or even pharmaceutical processes [12]. The intense interest in the ILs field raised in the last decade has been universal and interdisciplinary, and it has lead to advances both in fundamental knowledge and in technological applications [13]. 

Most literature references deal with the synthesis, physicochemical properties and characterization of ILs. Some recent reviews [5,6,7,8,9,10,11,12,13] have also focused on applications as solvent replacement, purification of gases, homogenous and heterogeneous catalysts, biological reactions media and removal of metal ions, electrolytes in batteries, lubricants, plasticizers, solvents and catalysts in synthesis, matrices for mass spectroscopy, solvents in the controlled synthesis of nanomaterials, extraction, gas absorption agents, etc. 

Applications in which ILs are used as novel engineering fluids are based on their solubility, their thermal and mechanical properties or some combination of these. These include their use as thermometric fluids in liquid-in-glass thermometers [7] or their use in a lunar telescope [14]. The negligible vapour pressure, substantial conductivities and thermal stability of ionic liquids make them ideal candidates for space applications, such as the compression of oxygen using an IL as operating fluid. For use in high pressure applications they should have low compressibility, adjustable gas solubility and good lubricating ability. 

A recent review [11], highlights advances of ILs as versatile *green* engineering liquids in a variety of industrial applications including heat transfer fluids, azeotrope-breaking liquids, lubricants, electrolytes, liquid crystals, supported IL membranes, etc. Interactions between two solvents such as ILs and supercritical CO_2_ are also being studied [10].

During the last decade, ILs have established themselves as promising advanced lubricants due to many of the same properties that make them useful as solvents and engineering fluids. The study of friction, wear and lubrication is the subject of study of the interdisciplinary field of tribology. A review by I. Minami in this same issue is dedicated to ionic liquids in tribology [13]. Closely related to the efficiency of ionic liquid as lubricants, are the surface interactions which take place at the material-IL interface. 

The present review will focus on the possibility of using ILs as lubricants in aqueous environments (water is a truly green lubricant in the room temperature range) or under severe conditions for which conventional lubricants fail, such as those found in spacecraft applications: ultra-high vacuum and extreme temperatures (from cryogenic conditions up to 500 ºC). We will also highlight the use of ILs in contact with difficult to lubricate materials such as the light alloys, due to their reactivity towards conventional lubricants, and in nanotechnology, where IL molecules act as surface modifiers of nanoparticles and nanotubes, or in lubrication of MEMS or NEMS systems, and nanocomposites. 

Tribosystems are present in all mobile mechanisms, from automotive, industrial, domestic, to aerospace applications. In tribosystems, one material slides against another or against itself. Even in this latter case, the geometry of the counterparts can be different and the contact is then asymmetric. For each combination of materials and contact configurations, the tribological variables include normal applied load, sliding distance, speed and frequency, temperature (ambient and contact temperature), atmosphere, moisture, etc. The geometry of the contact and the contact pressure change when one or both materials are worn during the sliding process. When wear debris are trapped at the contact zone, a third body is formed which can protect against further material removal or increase the surface damage by abrasion due to ploughing.

Lubricants are used to control friction and wear preventing direct contact between the surface asperities of the materials and lowering the contact temperature. Lubricant formulation is based on a mineral or synthetic base oil or grease with a mixture of different additives to control viscosity, surface interactions, corrosion and increase load carrying capacity, thermal diffusivity and service life. However, commercial lubricants are derived form petroleum, present environmental problems and are not suitable for many materials and conditions. The need for new effective, environmentally friendly lubricants is clear, as the losses due to friction and wear of materials can be estimated around a 1.5% of the gross domestic product (GDP) of industrialized countries [15]. 

## IL as Neat Lubricants

ILs based upon the 1-*n*-alkyl-3-methylimidazolium cation were first reported in 1982 by Wilkes *et al*. [16]. In 1992, Wilkes and Zaworotko [17] developed an air and water stable ionic liquid based upon the tetrafluoroborate anion. These water stable ILs were the first ones selected as lubricants. 

The field of IL lubrication was effectively started in 2001 by Liu *et al*. [18]. They studied alkyl-imidazolium tetrafluoroborates in steel/steel, steel/aluminium, steel/copper, steel/SiO_2_, steel/Si(100), steel/sialon and Si_3_N_4_/sialon ceramic contacts, showing excellent friction reduction. 

**Figure 2:**
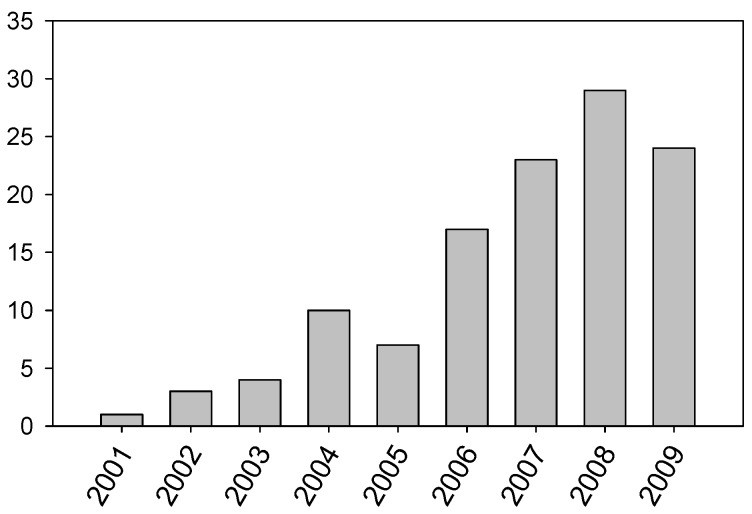
Evolution of the number of scientific papers published on the use of ILs in lubrication.

The interest of these results has steadily increased the number of scientific papers published each year (Figure 2) only on IL lubrication until June 2009, according to the Thomson ISI Web of Knowledge database. 

From the first works [18,19,20,21], the good lubricating performance of ILs was attributed to the surface interactions and tribochemical processes, which take place at the interface. Adsorption of IL molecules and formation of surface layers containing B_2_O_3_ and BN was proposed on the basis of X-ray photoelectron spectroscopy (XPS) analysis. 

The real time *in-situ* formation of FeF_2_ by tribochemical reaction between 1-ethyl-3-methyl-imidazolium tetrafluoroborate ([EMIM]BF_4_) and steel at room temperature has been demonstrated [22]. 

Most works on IL lubrication refer to steel-steel contacts, which are the most common. The main results obtained are reviewed by Minami [13]. Industries where the tribological considerations are critical are those of transport, where conventional alloys are being substituted by light alloys, and aerospace applications where severe conditions such as extreme temperatures, corrosive environments or high vacuum predominate. These are the areas where ILs can fill a gap. In what follows, we will review some of the main results obtained in these fields.

## ILs as Lubricants of Light Alloys

A major goal of the present research in tribology is to find effective lubricants for reactive light alloys such as magnesium [23,24,25,26,27,28,29], aluminium [30,31,32,33,34,35,36,37,38,39,40] and titanium [41]. These alloys are substituting conventional iron-based materials due to their lower densities, higher specific resistance and the ability to form corrosion-protective surface layers. However, surface interactions with IL molecules could also promote corrosive attack. 

Although several works have been dedicated to study the interactions between imidazolium based ionic liquid and reactive magnesium alloys [23,24,25,26,27,28,29], to our knowledge, the use of ILs as lubricants of magnesium alloys has not been reported.

The development of protective surface films on a magnesium alloy has been described [27]. The film results from the reactivity between magnesium and the IL and improves the corrosion resistance of the alloy. It remains to be studied if it could also provide higher wear resistance.

Titanium exhibits outstanding corrosion resistance in a wide variety of environments, specially oxidizing, neutral and inhibiting reducing media. The corrosion resistance of titanium is due to the formation of a protective and self-adherent oxide film. Nevertheless, this film is not stable in reducing acids and titanium is severely attacked.

Recently [38], ammonium and imidazolium ILs were studied as lubricants of titanium, finding that for both types of cations, a longer side chain gave better antiwear protection for titanium. At room temperature, the best performance was obtained for 1-methyl-3-benzylimidazolium chloride ([BzMIM]Cl), while at 100 ºC, the lowest friction and wear values were obtained for 1-hexyl-3-methyl-imidazolium hexafluorophosphate ([HMIM]PF_6_). Again, the lubrication results are related to tribochemical processes. Tetrafluoroborate imidazolium ILs decompose at the metal-metal contact producing severe tribocorrosion, with formation of metallic fluorides, boron carbide and boron oxide. In contrast, under the conditions studied, the hexafluorophosphate lubricant forms adsorbed layers on the titanium surface and a phosphate-containing protective layer on the steel counterface.

Among the light alloys, the most studied are aluminium alloys due to their wide range of application in sliding components, particularly in the automotive industry.

A series of room temperature ionic liquids with phosphonyl groups on the imidazolium cation, 1-(3'-*O,O*-diethylphosphonyl-*n*-propyl)-3-alkylimidazolium tetrafluoroborate, were prepared [31] and evaluated as promising lubricants for the contact of aluminum on steel. The new functionalized imidazolium ILs with lateral phosphonyl groups showed superior tribological performance as neat lubricants of steel/aluminium contacts than nonfunctionalized alkylimidazolium derivatives, due to surface interactions through the phosphorus atoms. The formation of films composed of bridged complex compounds, metal fluorides, nitrogen oxide, B_2_O_3_, BN, and FePO_4_ on the rubbed surfaces was proposed. 

**Figure 3:**
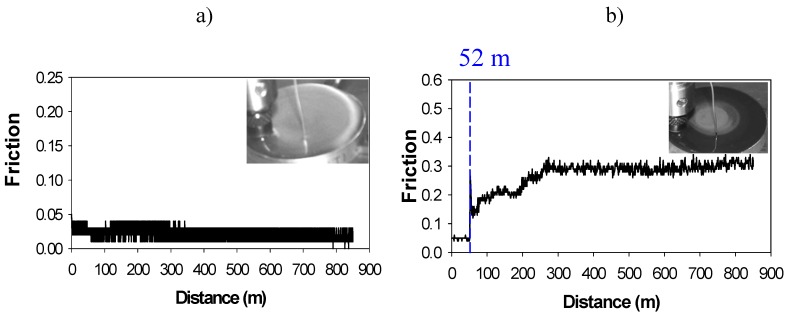
Friction records and appearance of the aluminium surface covered by IL after the test**:** a) Low constant friction in the absence of tribocorrosion; b) Friction increase due to tribocorrosion.

The influence of the alkyl chain length on the cation and that of the anion on the lubricating ability has been studied for 1-*n*-alkyl-3-methylimidazolium ILs in steel/aluminium contacts [32]. Real-time sharp friction increments (Figure 3) related to tribochemical processes have been observed for tetrafluoroborate and hexafluorophosphate ILs, at room-temperature and at 100 ºC. 

Wear scar surfaces are oxidized to Al_2_O_3_ and wear debris contain aluminium and iron fluorides, in the case of tetrafluoroborate ILs. For hexafluorophosphate ILs, the steel surface is covered with a phosphorus-containing tribolayer. A change of anion reduces friction and wear, but the lowest values are obtained by increasing the alkyl chain length on the imidazolium cation. 

Although imidazolium ILs constitute the more extensively studied family of IL lubricants, other cations have been also used in a more limited number of studies, with promising results in the case of ammonium derivatives [37,38]. A quaternary ammonium ionic liquid crystal was shown to have superior performance under severe contact conditions in steel/aluminium lubrication than neutral liquid crystals [39]. A series of trialkylammonium ionic liquids were synthesized by neutralization and metathesis reactions, and have demonstrated promising lubricating properties as neat lubricants or lubricant additives, particularly for use with difficult to lubricate metals like aluminium.

Different aluminium alloys have been studied in the presence of trialkylammonium and imidazolium ILs at room temperature and at 100 ºC [37,38]. Results depend on the aluminium alloy–IL surface interactions, thus showing that each material-IL pair needs to be evaluated independently before general conclusions can be reached. Surface interactions of alkylimidazolium ionic liquids (ILs) with aluminium alloys have been studied [40] by immersion tests in seven neat ILs, six imidazolium ILs and one pyridinium derivative. Immersion tests have also been carried out in 1 wt.% and 5 wt.% solutions of 1-ethyl-3-methyl-imidazolium tetrafluoroborate ([EMIM]BF_4_) in water. No corrosion of Al by the neat ILs is observed. The highest corrosion rate for Al in water is observed in the presence of a 5 wt.% [EMIM]BF_4_, probably due to hydrolysis of the anion and formation of aluminium fluoride. 

Erosion-corrosion processes have been studied for three aluminium alloys in a 90 wt.% [EMIM]BF_4_ solution in water in the presence of α-alumina particles [40]. The erosion-corrosion rates are around 0.2 mm/year or lower, and increase with increasing copper content, thus showing that the interactions with ILs need to be determined for each set of conditions, considering the material and IL composition. 

## ILs as Lubricants under High Vacuum

Liu *et al*.’s [19] preliminary studies included ionic liquid lubrication at 100 ºC and *in vacuo*, finding that the wear rate of steel was lower *in vacuo* in the presence of an alkylimidazolium tetrafluoroborate, thus anticipating that ILs could provide effective lubrication in space applications.

Alkylimidazolium hexafluorophosphate ILs were studied as lubricants of steel/steel contacts under vacuum [42,43], showing superior performance than conventional oils, even modified by antiwear additives. The formation of FePO4 and FeF2 as a result of the tribochemical reaction of the IL and the steel surface was determined by XPS.

Tribological characteristics of two imidazolium-based ILs, 1-hexyl-3methylimidazolium tetra-fluoroborate ([HMIM]BF_4_), and 1-hexyl-3-methylimidazolium hexafluorophosphate ([HMIM]PF_6_) were investigated under high vacuum conditions [42]. Viscosity-temperature characteristics and thermogravimetric characteristics of these compounds were also investigated. Imidazolium-based ILs have relatively good viscosity-temperature characteristics that are comparable to those of multiply-alkylated cyclopentane. Thermogravimetric results showed that ILs have high thermal stability and low vapour pressure. ILs showed low friction and low wear rate under high vacuum conditions, and high load-carrying capacity was observed. 

## ILs as Extreme Temperature Lubricants

The surface chemistry of IL-lubricated steel in sliding contact at temperatures from room to 300 ºC has been studied [44]. Some iron samples were oxidized to Fe_2_O_3_ and Fe_3_O_4_ prior to treatment with ionic liquids. The metallic and oxidized samples were then reacted with ionic liquids at elevated temperatures. Chemical analysis revealed corrosion of the surface due to reaction between the ILs and the steel/iron substrates. 

A series of new polyethylene glycol functionalized dicationic ILs with alkyl or polyfluoroalkyl substitutents has been prepared [45]. These ILs show high thermal stability and good lubricity. In general, imidazolium based dicationic liquids have higher degradation temperature (Td>400 ºC) than their triazolium analogues. The introduction of polyfluoroalkyl groups boosts antiwear properties but also leads to a decrease in Td. The dicationic ILs also exhibit excellent tribological characteristics even at 300 ºC, which suggests their potential as high temperature lubricants. 

A new class of dicationic ionic liquids with a bridging moiety, such as a polyalkylether, polyfluoroalkyl, 1,4-bismethylenebenzene, or 1,4-bismethylene-2,3,5,6-tetrafluorobenzene, between the alkyl-substituted imidazolium rings has been described [46]. Their properties were modified by varying the linker chains and/or alkyl substituents on the imidazolium ring. These new ionic liquids (except with PF_6_^-^ as anion) displayed outstanding tribological properties in temperature ramp tests by performing very well at 300 ºC, thus meeting one criterion for high-temperature lubricants. 

The first study of ILs as steel-aluminium lubricants under a wide range of temperature conditions (‑30, 100, and 200 ºC) [33] compares the tribological performance of the imidazolium ionic liquids 1-hexyl- and 1-octyl-3-methylimidazolium tetrafluoroborates ([HMIM]BF_4_ and [OMIM]BF_4_, respectively) with that of a mineral oil and the synthetic ester propylene glycol dioleate in aluminium-steel contacts. ILs show lower friction and wear values than conventional oils at all temperatures. 

The lubricating performance depends on thermal stability, polarity of the molecules, their ability to form ordered adsorbed layers and the tribocorrosion processes which take place at the interface. While the conventional oils fail above 150 ºC due to thermal decomposition, the longer alkyl chain [OMIM]BF_4_ presents higher viscosity values than [HMIM]BF_4_, and provides an effective surface separation at all temperatures, and only shows friction and wear increments at -30 ºC in the presence of water, due to severe abrasion by ice crystals. 

**Figure 4:**
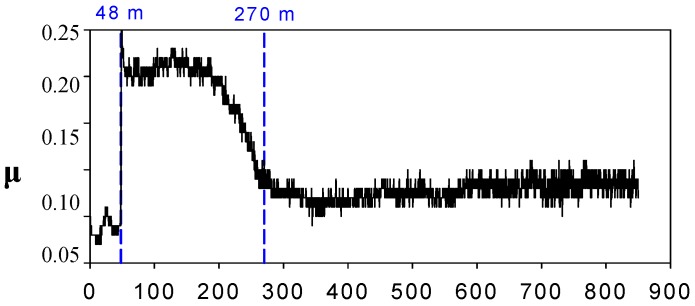
Friction (μ) vs. sliding distance (m) for steel-aluminium contact lubricated with [HMIM]PF_6_ at 200 ºC [33].

The more polar molecules of the shorter alkyl chain [HMIM]BF_4_ produce severe wear at 200 ºC due to the formation of aluminium fluoride wear debris by tribocorrosion reactions. The time for tribocorrosion to take place, has been determined from friction increments (see Figure 4) and wear debris generation. 

## Surface Interactions

In order to understand the complex surface chemistry which takes place at the materials-IL interfaces, most of the reported tribological works contain detailed surface interactions studies by XPS, particularly in the case of ceramic and metal lubrication with imidazolium hexafluorophosphates and tetrafluoroborates.

The general process which takes place includes oxidation, dissociation of the anions, formation of metallic phosphates, oxides and fluorides, and precipitation of ceramic phases such as boron and phosphorus fluorides, boron oxide, boron carbide, and boron nitride. Decomposition of the imidazolium cation with formation of nitrogen oxides has also been proposed. During the wear process, nascent metallic surfaces are produced which could enhance the metal-IL reactivity and favour the process of IL decomposition.

When the formation of bridge complexes between the surfaces and the IL molecules is favoured due to the presence of substituents with electron-donor atoms, the adsorbed IL layers are more stable and protect the surface.

For effective lubrication, these stable adsorbed tribolayers must combine with the presence of flexible units. The length of these units is a critical parameter in the antiwear properties as it determines the polarity of the IL. 

Interestingly, IL additives can be as effective, or even more effective than neat ILs in reducing friction and wear. This can be explained by the fact that a low proportion of IL (around 1wt.%) contains a number of IL molecules which is enough to form stable adsorbed layers on the surface, but not to produce the severe tribocorrosive attack found for fluorine-containing short alkyl chain ILs.

Metallic alloys which form protective oxide layers such as aluminium, titanium, nickel, etc. are resistant to corrosion by immersion in neat IL, but when they are sliding against a more reactive counterface such as the bearing steel found in many moving parts, the decomposition of the IL is promoted due to the reaction with iron. The decomposition products can then react with the more corrosion resistant materials, giving rise to sharp friction and wear increases, as we have seen in Figure 3(b) and Figure 4.

## Theoretical Studies and Models for IL Lubrication

A scaling concept based on relaxation theories of the liquid state has been proposed [47] to provide a general framework describing the dependency of viscosity with pressure and temperature. The viscosity-pressure coefficient was expressed in terms of a state-independent scaling exponent. This scaling factor was determined empirically. New equations for the pressure- and temperature-viscosity coefficients were derived for elastohydrodynamic lubrication. These relations can be applied over a broad range of thermodynamic conditions. The fluids considered include several ionic liquid compounds selected to represent molecules of different sizes and with diverse intermolecular interactions. 

**Figure 5:**
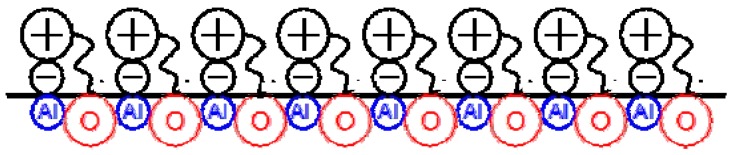
IL-Al_2_O_3_ surface interactions.

The interactions between selected ionic liquids and either an aluminium oxide or a silicon nitride surface have been modelled using semi-empirical methods [48]. The ionic liquids include a series of cationic imidazolium derivatives with hexafluorophosphate anion. The tribological properties of these ionic liquids are modelled using a smooth alumina surface (Figure 5). 

The ILs are allowed to form a complex with this surface and the enthalpies of complex formation are seen to correlate with the tribological properties of the seven ionic liquids. The ILs are also complexed with a hexagonal silicon nitride surface and their tribological properties are predicted on the basis of complex formation enthalpies. 

Nanometer-scale structuring in ILs has been observed from theoretical simulations [2]. Segregation is observed for ILs of the 1-alkyl-3-methylimidazolium family with alkyl side chains in the cation longer than or equal to butyl. The alkyl chains aggregate in non-polar domains that permeate a three-dimensional network of ionic channels formed by the charged parts of the ions, giving rise to medium-range ordering. 

In a very recent study on the frictional dynamics of alkylsilane monolayers on SiO_2_ [49] the simulations indicate that the molecules of the IL 1-butyl-3-methylimidazolium nitrate ([BMIM]NO_3_) could incorporate into a damaged area of the self-assembled monolayer (SAM) coating and restore the tribological properties of the film. 

## Ionic Liquids as Lubricant Additives

### ILs in water

One remarkable characteristic of ILs is their organization in solution, with polar and non-polar domains [1,2,3] and their miscibility with polar and non-polar solvents. ILs have been used as lubricating additives both in water and in lubricating oils.

In the first work on the use of ionic liquids as lubricants, Liu *et al*. [18] already included a reference to the interactions of the IL lubricants studied with water, stating that the addition of a small amount of water (≤ 5wt%) to the ILs could help to improve the antiwear behavior for some metal-metal contacts.

When used as additives in water [50,51], ILs reduced the running-in period (the initial period of high friction) of ceramic-ceramic sliding contacts. The proposed mechanism was the initial smoothening of the surface by mechanical wear, the formation of BFx and PFx films on the surface and the creation of an electric double layer of IL molecules, which increases the local viscosity near the surface and the load carrying ability and lowers friction and wear.

The lamellar microstructure composed by the non-ionic surfactant polyoxyethylene laurylether, the room temperature ionic liquid 1-butyl-3-methylimidazolium hexafluorophosphate ([BMIM]PF_6_) and water has been investigated [52]. The lubrication properties of the lamellar mesophase were determined to illustrate their relationship with the microstructure. The results showed that the structural strength of the lamellar phase is enhanced with increasing amount of the non-ionic surfactant, so the anti-wear capacity of the lamellar phase is improved. 

### ILs as mineral oil additives

**Figure 6:**
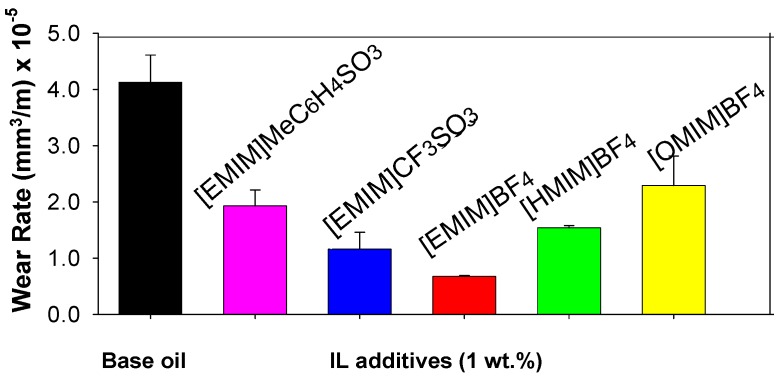
ILs as additives in steel-aluminium lubrication.

1-*n*-Alkyl-3-methylimidazolium ILs, with different side chain lengths on the cations, and different fluorine-containing anions have been studied as mineral oil additives [36]. Figure 6 shows the effect of the addition of a 1wt.% proportion of imidazolium ILs to a lubricant oil on the wear rate of aluminium at room temperature. 

For the same anion [BF_4_^-^], the more effective neat ILs lubricants were those containing cations with longer alkyl chains: octyl- ([OMIM^+^]) better than hexyl- ([HMIM^+^]) and better than ethyl- ([EMIM^+^]). 

In contrast, when used as additives, the more effective wear reducing ILs are those with shorter alkyl chains: [EMIM^+^] better than [HMIM^+^], better than [OMIM^+^], as can be observed in Figure 6. For the same cation ([EMIM^+^]) and different anions, the wear rate of aluminium decreases in the order: MeC_6_H_4_SO_3_^-^ > CF_3_SO_3_^-^ > BF_4_^-^. 1 wt.% IL additives also show good lubricating performance at 100 ºC. 

Wear rate is the material volume loss, in this case aluminium alloy, by unit of sliding distance.

### ILs as synthetic oil additives

1-*n*-Alkyl-3-methylimidazolium ILs, with different side chain lengths and the fluorine-containing anions hexafluorophosphate [PF_6_^-^], tetrafluoroborate [BF_4_^-^] and triflate [CF_3_SO_3_^-^] have also been studied as 1 wt.% additives of the synthetic ester propylene glycol dioleate in steel-aluminium contacts at 25 and 100 ºC [35]. At 100 ºC, all additives reduce both friction and wear with respect to the base oil. Friction coefficients for IL additives are similar or lower than for neat ILs, while wear rates for 1 wt.% ILs can be several orders of magnitude lower than those for neat ILs. In this case the contact conditions for optimum lubrication were the same for all additives independently of composition. 

Very recently [53], dicationic bis(imidazolium) ILs with the same long side-chain substituted cation and different anions were evaluated as additives in polyethylene glycol at room temperature. Results showed that they could effectively reduce the friction and wear of steel-steel sliding pairs compared with the base oil without additives. The dicationic IL with [(CF_3_SO_2_)_2_N^-^] anion showed better antiwear properties for an optimum concentration of 3 wt.%. 

**Figure 7:**
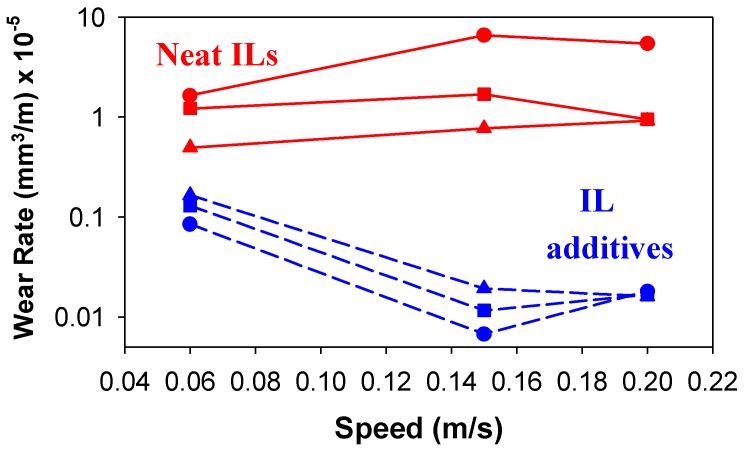
Comparative antiwear performance of [EMIM^+^] ILs as neat lubricants and 1 wt.% additives [36].

IL additives not only reduce the friction and wear with respect to base oils, but they show better performance than neat ILs, as shown in Figure 7 [36] for steel-aluminium at room temperature, particularly for a determined sliding velocity which improves IL miscibility with the base oil.

The excellent tribological properties of IL additives are attributed to the formation of physically adsorbed films, formation of tribochemical products during friction without tribocorrosion, and good miscibility with the base oil.

### Additives of IL lubricants

In order to reduce the corrosive attack of the more reactive ILs, particularly those containing fluorine anions, several studies have attempted their modification by wear-reducing and corrosion inhibitor additives [54,55,56,57,58]. 

Benzotriazole (BTA) was studied as lubricant additive of ILs because of its similar molecular structure [54,55,56]. BTA can greatly improve the tribological behaviour of ILs with the hexafluorophosphate anion for the steel/Cu-Sn alloy sliding pair due to corrosion inhibition. Strong interaction between BTA and the surface of Cu alloy was proposed to account for the excellent anti-wear and anti-corrosion improvement capability. In particular, a protective film containing [Cu(C_6_H_5_N_3_)] and Cu_2_O has been proposed upon the basis of XPS analysis.

Diimidazoliumalkylene hexafluorophosphate ILs were synthesized and doped with BTA showing excellent anti-wear ability. The worn surfaces and chemical nature of the boundary films generated on the metal surfaces were analyzed. Results showed slight abrasion on the worn surfaces and XPS results indicated the formation of FeF_2_, FeF_3_, Fe_3_O_4_, and FePO_4_ by the tribochemical reactions of ionic liquid with iron during the sliding process. Tricresylphosphate (TCP) and dibenzyldisulfide additives have been found to improve antiwear properties of ILs to some extent. Minami *et al*. [57] have described the first additives that improve the tribological properties of ionic liquids. Tetraalkylammonium and tetraalkylphosphonium salts of *N*-protected aspartic acid was dissolved in 1-alkyl-3-methylimidzolium bis(trifluoromethylsulfonyl)imide. They prevented wear remarkably and reduced friction considerably. A model of boundary film composed of liquid clathrate structure was proposed. 

Addition of 1% TCP to ionic liquids [58] rapidly establishes a tribofilm and reduces the wear volume by 64% compared to the same test for the neat ionic liquid or neat TCP. Addition of 1% TCP and 1% IL to a base oil also establishes a substantial tribofilm and reduces wear volumes compared to the base oil with 1% TCP alone or the base oil with 1% ionic liquid alone. 

ILs show promise as neat liquid lubricants by establishing a tribolayer chemically adsorbed to the steel surfaces, but they are not as effective as a reference hydrocarbon lubricant in reducing wear due to tribocorrosion. The fluorine-free ILs investigated were not as effective as those containing fluorine. The addition of ILs to grease, gave a substantial improvement in performance, which indicates a synergistic interaction with the additives present in the formulated grease. There is also clear evidence of a strong synergistic effect between ILs and TCP, but the nature of the synergy between ILs and TCP requires further investigation. 

In the preceding sections we have reviewed the results obtained in conventional materials lubrication and conditions. In the following sections, some special applications and systems will be addressed. 

### MEMS lubrication

**Figure 8:**
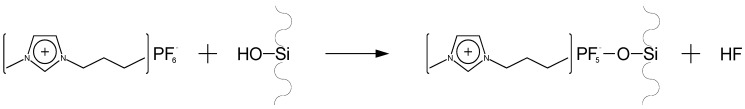
Proposed model for the attachment of [BMIM]PF_6_ to silicon [59].

Palacio and Bushan [59] have studied the formation of ultra-thin wear resistant IL films for MEMS/NEMS applications, proposing a model for the attachment of [BMIM]PF_6_ to the silicon substrate (Figure 8).

Contact failures in microelectromechanical system (MEMS) switches, particularly during hot switching, prevent their widespread use. A nanoparticle-liquid (NPL) lubricant is synthesized and deposited on MEMS switch contacts as a nanotechnology based lubricant [60]. NPLs are monolithic hybrid materials comprised of an inorganic nanosized metallic core and an organic low viscosity corona. The NPLs used contain Au or Pt nanoparticles as the core and a mercaptoethanesulfonate IL as the corona. NPLs exhibited improved electrical performance and durability as compared to uncoated and self-assembled monolayer-coated switch contacts. These results show that NPLs could have an excellent potential as surface modifiers/lubricants for MEMS switch contacts. 

When MWCNTs are dispersed within a hexagonal lyotropic liquid crystals (LLCs) formed in the IL, ethylammonium nitrate [61], polarized optical microscopy images combined with small-angle X-ray scattering (SAXS) results indicate that the MWCNTs are well-dispersed and that the introduction of MWCNTs does not destroy the structure of hexagonal LLCs. The increase of d spacing demonstrates the integration of MWCNTs within the cylinders of the hexagonal LLCs. 

The tribological properties were explored in order to extend the applications of MWCNTs-LLC composites in ILs as lubricating materials, although only macroscale friction and wear tests have been conducted on these materials. The rheological measurements indicated that MWCNTs-LLC composites are highly viscoelastic and that the apparent viscosity is enhanced by the presence of the MWCNTs. 

ILs are considered as lubricants for micro/nanoelectromechanical systems (MEMS/NEMS) [59,62,63] due to their excellent thermal and electrical conductivity. Evaluating the nanoscale tribological performance of ILs when applied as thin film on a substrate is a crucial step to understand how they can efficiently lubricate MEMS/NEMS devices. 

The adhesion, friction and wear properties of two ionic liquids, 1-butyl-3-methylimidazolium hexa-fluorophosphate ([BMIM]PF_6_) and 1-butyl-3-methylimidazolium octylsulfate ([BMIM][OctylSO_3_]), applied on Si(100), was investigated using atomic force microscopy (AFM). Wear at ultra-low loads was simulated and the lubricant removal mechanism was investigated using AFM-based surface potential and contact resistance techniques. Thermally treated coatings containing a mobile lubricant fraction were able to protect the Si substrate from wear compared to the fully bonded coatings, and this enhanced protection is attributed to lubricant replenishment. 

MEMS devices coated with a thin film of ionic liquid showed significant improvement in wear life [64,65]. A method that is based on atomic force microscopy (AFM) with a liquid cell has been developed [64] to study friction and wear properties of surfaces lubricated with ILs. The friction and wear data obtained for these tests showed good correlation with the failure life span of lubricated microelectrical mechanical systems (MEMS) motors. 

The understanding and the improvement of wear of the tip during its contact with the ferroelectric materials is critical, particularly at the high scanning velocities needed for high data rate recording in the operating environments. To this end, adhesion, friction and wear experiments have been performed [66] using Pt-Ir coated tips sliding against unlubricated and lubricated lead zirconate titanate films. Two lubricants were used: perfluoropolyether and ionic liquid. The Pt-Ir tips are shown to exhibit lower wear against the lubricated ferroelectric film. 

## IL-modified Nanostructures

As it was anticipated [67], the unique combination of polar structure and the strong hydrogen bond interactions has open a new area of application of ILs as reaction media for the synthesis of nanostructured solids. 

**Figure 9:**
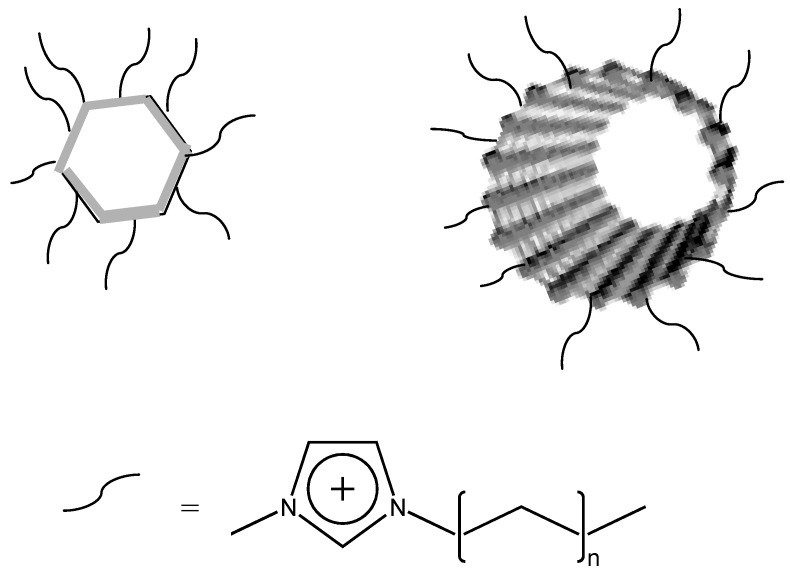
IL modified nanoparticles and nanotubes.

Recent reports have described the use of ILs as solvents for the synthesis of nanoparticles of palladium, platinum, iridium, gold, single-crystalline tellurium nanorods and nanowires by using ILs as solvents, and in surface functionalization of nanoparticles [67,68]. IL-modification (Figure 9) produces liquid-like organic salts and allows to enhance processability, and a variety of ZnO nanoparticles morphologies has been described in ILs media. 

Fukushima *et al*. [69], found that single-walled carbon nanotubes (SWCNTs) can be readily dispersed in imidazolium IL by mechanical milling. The IL interacts with SWCNT (Figure 9) through van der Waals interactions and shields the strong π-π stacking interaction between SWCNTs, thus stabilizing their dispersion [70].

The new SWCNT-IL bucky gel has been used in the preparation of a new plastic actuator, and polymerization of the gel produces a mechanically reinforced electrical and thermal conductive soft material [71,72].

IL/multi-walled carbon nanotubes (MWCNTs) have been prepared by chemical modification The new IL/MWNTs composite was evaluated as lubricant additive in IL due to their excellent dispersibility. It has been found that IL/MWCNTs showed good friction-reduction and anti-wear properties [73].

## Polymer Nanocomposites

Because ILs are stable liquids over a wide temperature range, they offer technological advantages over some chemicals used in their liquid phase, such as plasticizers, where polymer flexibility can be enhanced. Common problems with plasticizers include evaporation and leakage from the surface, instability at high temperatures, lack of lubrication at low temperatures, migration within the polymer, and toxicity. Systems studied include polymethylmetacrylate (PMMA) plasticized with hexafluorophosphate [PF_6_^-^] ILs [8]. 

Ionic liquids as plasticizers might produce though flexible polymers. The lubricating ability of ILs has also been shown in polymers, both as external lubricants of polymer/steel systems and as polymer additives [22,74,75,76]. 

The tribological properties of polyamide (PA6) containing the ionic liquid 1-hexyl-3-methyl imidazolium hexafluorophosphate ([HMIM]PF_6_) as 3 wt.% additive dispersed in the polymer matrix have been studied at variable temperatures. The IL-modified polymer maintains a low friction coefficient in the whole range of temperatures with values similar or even lower to that obtained when IL is used as external lubricant. [HMIM]PF_6_ has been added [76] to the polycarbonate (PC) nanocomposite (PC+0.5 wt.%ZnO) in a 1.5 wt.% proportion, to obtain PC + 0.5%ZnO + 1.5%IL. 

**Figure 10:**
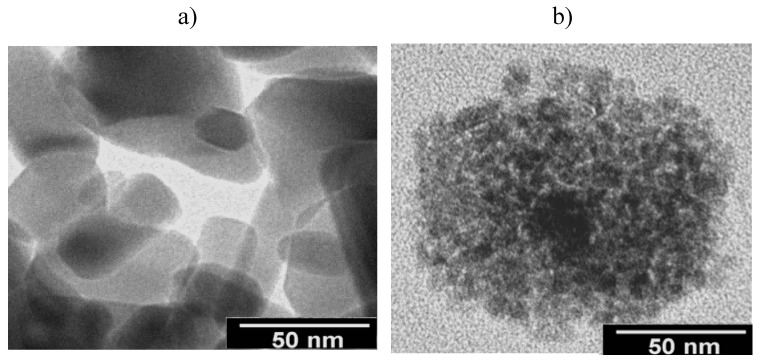
TEM micrographs of: a) Neat ZnO nanoparticles; b) IL-modified ZnO nanoparticles within PC matrix.

The new nanocomposite shows a 80% friction reduction and a wear reduction of nearly two orders of magnitude with respect to PC + 0.5%ZnO. This good tribological performance is attributed to the improvement in ZnO dispersion within the PC matrix due to surface modification (Figure 10) of the nanophase by the IL.

## Conclusions and Outlook

The results reported here show that ILs are a family of new high performance lubricants [77] which may find commercial and technological applications where other lubricants are not suitable or fail to prevent friction, wear and surface damage of materials. 

An adequate IL lubricant should be found for use in environments such as high vaccum, extreme temperatures and high pressures. To achieve this, a larger number of ILs should be tested under variable conditions. The surface interactions and tribochemical reactions of ILs with the sliding counterparts need to be thoroughly examined in order to ensure the formation of protecting tribofilms and to avoid corrosion. 

The strategies followed so far to avoid severe tribocorrosion, particularly in the case of fluorine-containing ILs, include the use of reactive ILs as lubricant additives rather than as neat lubricants, and the addition of corrosion inhibitor additives to IL lubricants. The synergistic effect and long-term stability of these mixtures need to be further investigated. 

The IL-lubricated sliding contacts that have been reported are mainly steel-steel, steel-aluminium, steel-ceramic and ceramic-ceramic. More information is needed about the feasibility of the use of IL lubricants in contact with cryogenic or high temperature materials. 

Very limited work has been reported on polymer lubrication by ILs and polymer-IL interactions in new nanocomposites. Promising results have been obtained in the used of IL–modified nanophases in lubrication, but further tribological studies should follow.

Finally, a complete model of surface interactions and IL organization and structure is still to be developed. Many questions remain to be answered, but this will be the subject of the efforts of the growing number of researchers that are incorporating to this research area. 

For commercial implementation ionic liquids still need to meet a number of requirements such as cost, stability and toxicity and environmental considerations. Some attempts are already been made to develop ILs from cheap starting materials [78].

Most ILs presented here contain halogen atoms and may release toxic and corrosive hydrogen halides to the environment [79,80]. New halogen-free ILs are being developed to avoid corrosion and toxicity [81], but few toxicological and/or ecotoxicological data are available for ILs until now.
